# Pennsylvania Hospital

**Published:** 1838-07-18

**Authors:** Henry H. Smith

**Affiliations:** Resident Surgeon


### CLINICAL REPORTS.

### PENNSYLVANIA HOSPITAL.

[Reported by Henry H. Smith, M.D., Resident Surgeon.]

1. *Successful operation for Lithotripsy, in a boy, thirteen years of age, by* J. Randolph, M. D., *Surgeon to the Hospital.*

George House, æt. thirteen years, was admitted into the hospital on the 30th of March, 1838, for stone in the bladder. Eleven years since, he suffered from the same complaint, for which he was cut by Dr. Randolph. The stone was removed entire; no water was passed by the cut after the first day, and the wound united by the first intention. On his admission to the hospital, he suffered frequently from fits of stone, which were relieved by anodynes and hip-baths.

On the third of April he was sounded by Dr. Randolph, and the stone distinctly heard. He was kept on light nutritious diet. After the sounding, his urine had a small quantity of sabulous matter in it. The boy had been spoiled by indulgence at home, and the sight of the instruments caused him to cry. After two days, a metallic bougie was introduced, so as to dilate the urethra, and accustom it to the instrument; this was practised daily.

*April 8th*—A large sized bougie was introduced without much complaint by the boy. Urethra small for one of his age.

*April 10th*—Dr. Randolph, in the presence of Drs. Smith, Pepper, and Meigs, introduced a small sized instrument of Jacobson, and caught the stone immediately. The boy complained slightly, but his complaints evidently arose more from fear, than from any considerable pain, as, during the greater part of the time, he raised himself on his elbows, to observe the operation. The stone was easily broken on the first seizure, and again on the second; the boy was then put to bed, and ordered a hip-bath, and ten drops of laudanum.

*April 11th*—He has passed several fragments of stone; suffers much on making water; fecates with difficulty; ordered hops, boiled in vinegar, hot, to the perineum, in bags of flannel. Half an ounce of castor oil.

*April 13th*—Since last day, has passed three large pieces of stone, without much pain; great relief from applications; says he feels quite easy.

*April 16th*—Was sounded yesterday by Dr. RANDOLPH; stone broken into several fragments; has passed, since last day, several pieces of stone; suffers little at present; formerly he had several paroxysms in a day.

*April 17th*—Has passed a large piece of calculus, which caused much pain and some little fever; is otherwise doing well. Ordered pulv. Dover. gr. iv., ol. cinnam. gtt. ¼, twice a day.

*April 18th*—Dr. RANDOLPH operated in presence of the class; the stone was readily caught and crushed; the boy says the operation gave him little pain; ordered hip-bath, and laud. gtt. x., at bed-time.

*April 19th*—Rested well; is quite easy; passed several pieces of stone, and much sabulous matter; continue laudanum at night.

*April 22d*—Passed more stone; small piece fast in urethra; continue treatment; hip-bath and injection, &c.

*April 23d*—Penis much swollen and inflamed; stone still in urethra; continue treatment; inject eighteen drops laudanum; hip-bath, &c.

*April 24th*—Passed large piece, apparently the oval end of the stone; inflammation of penis disappearing; no pain; able to walk about, without inconvenience; urine clear.

*April 25th*—Has passed a large piece of central portion of stone last night; suffers no pain.

*April 27th*—Dr. RANDOLPH caught stone three times easily, and broke it fine; says it caused little pain.

*April 28th*—Passed much fine sand; doing well.

*April 30th*—Sounded by RANDOLPH; piece of stone detected; bladder nearly free.

*May 1st*—Passed a large piece in the evening, which caught in the urethra, near the orifice, and was removed by the forceps; able to hold his water four hours; urine tolerably clear; suffers little.

*May 3d*—The boy is comfortable; some deposit in the urine; ordered uva ursi ℥ss., carb. sodæ ʒj. in a pint of water, wine-glassful every three hours; walks about.

*May 7th*—Operated upon by R.; a piece which was at the neck of the bladder, was pushed back by the sound the instant it was introduced; caught stone at once, of considerable size; broke it twice.

*May 9th*—Has passed numerous pieces of stone; holds his water four hours; suffers little; walks about.

*May 11th*—Sounded yesterday; the stone in numerous pieces, several of which have been passed; urine quite clear; continue tea.

*May 13th*—The stone, in fragments, was caught four times; little pain; running in the yard in the afternoon.

*May 14th*—Easy; passed a great deal of stone, mostly sandy.

*May 20th*—Since the last date, the boy has been going about; has passed no more stone; suffers no pain; holds his water; sounded by Drs. RANDOLPH and SMITH; no stone felt.

*May 25th*—Sounded again, and nothing felt; his urine was tested, and proved acid.

*May 26th*—Discharged, completely cured.

---

2. *Case of incised wound of Abdomen,—stabbed May 7th*, 1838.

William Early, æt. thirty-four, admitted at a quarter before one o'clock, P. M., May 7th, 1838. Wound in right side, in false ribs, of two and a half inches in length; penetrated obliquely; probe entered four inches; bleeding freely on entrance; pulse weak and slow; face pale; extremities cool; slight flesh wound in groin close to artery; wound dressed with straps out of house; ordered laud. 30 gtt., one stitch through muscles and integuments, dry lint and compress to wound, tight bandage from pubes to chest, little weak wine water.

*Night*—Skin warm and dry; pulse stronger; complains of pain on breathing; bandaged to prevent action of diaphragm; laud. 80 drops.

*May 8th*—Pulse quick and tense, ninety-two in minute; respiration forty-four in minute; bowels not opened; passed water freely; no sickness of stomach; pain in pubic region on pressure; V. S. ℥xiii., cal. gr. ij., opii gr. j.; pills every three hours; cold drinks acidulated; neut. mist.

*Night*—Leeches, four dozen, to abdomen, and warm poultices.

*Ten o'clock*—Pulse strong; V. S. ℥xvi.

*May 9th*—Unable to pass water; introduced catheter; bowels opened by injection, slightly; some tympanitis; continue treatment.

*Night*—Pulse active and strong; repeat leeching.

*May 10th*—Skin moist; less pain in abdomen; gums touched; stopped cal.; ordered dose oil, and opii gr. j., every three hours; gruel diet; continue poultice; injection at night of assafœt.

*May 11th*—Abdomen soft; slight pain on pressure; slight tympanitis; oil operated slightly; repeat oil; continue treatment.

*May 12th*—Bowels opened freely; slept well; tongue clean; pulse ninety-six, and soft; no tympanitis; dressings removed; wound partly united; dressed with strips and lint; continue treatment.

*May 13th*—No pain; countenance good; pulse soft; skin moist; wound re-dressed; slight serous discharge; little pain any where on pressure; slight pleuritic pain; ordered mist. neut. ℥vi., ant. tar. gr. ss., a table-spoonful of each.

*May 14th*—Bowels freely opened by mist.; pulse good; skin soft and moist; considerable discharge from wound—think from an abscess in lower portion of wound, external to ribs; dressed with plaster and compress.

*May 25th*—Walking about.

---

3. *Case of compound comminuted fracture of both bones of leg; died in seven days.*

Thomas Gillet, æt. thirty-six, was run over by a mail waggon, heavily loaded, on the 7th of May, at four o'clock, and was immediately brought to the hospital. The right leg was fractured in the tib. and fibula, below the knee; a longitudinal fracture of tibia of three inches in length; piece protruding at wound three inches above ankle, with oblique fracture of both bones at same point; great effusion of blood under fascia, near knee; bad constitution; intemperate; drinks over half a pint daily; dry lint, compress over wound, which bleeds freely; leg loosely bandaged, in fracture box and lead water cloths; laudanum thirty drops, at once, and one hundred and ten at bedtime.

*May 8th*—Slept none; complains of pain; starts at every thing; incipient mania a potu; laudanum; soup with pepper; leg untouched.

*May 9th*—Dozed a little; leg less painful; dressing removed; wound has bled freely; bone projecting from it; loose from tibia, but muscles adherent; great effusion, as high as knee, of blood; great edema; wound enlarged with bistoury, and free access given to coagulated blood; tendency to sphacelus, as high as knee; dressed with large warm poultice, covering whole leg; fracture box; morph. sulph. gr. ss., every four hours; full diet and porter.

*Night*—Slept some during day; no pain in limb; continue treatment.

*May 10th*—Mortification has extended half way to groin; leg much swelled; pulse feeble and quick; skin cold; face bronzed; tongue like a chip; bowels opened by injection; ordered fomenting poultice to whole leg, flaxseed to thigh, quin. gr. j., every hour, full diet, and brandy punch in addition to former treatment.

*May 11th*—Mortification has reached groin; pulse exceedingly feeble and fluttering; skin cold and clammy; facies hippocratica; continue dressing to leg; continue treatment, with morph. every four hours; repeat punch and porter.

*May 12th*—Vomits every thing; extremities cold; pulse scarcely perceptible; ordered ammon. mist. and brandy by injection; same dressing.

*May 13th*—Sinking; continue treatment.

*May 14th*—Died this morning, at one A. M.

---

*List of Accidents, admitted into the Pennsylvania Hospital, from June 27th to July 11th*, 1838.

One case of lacerated wound of the hand, caused by the bursting of a pistol: a piece of wadding lodged in the hand, and was removed—wound poulticed, and the hand placed on a splint. Pus travelled under the fascia, which was freely opened; and the wound is now doing well. One case of lacerated wound of the head—poulticed, and afterwards dressed with mucilage; now granulating kindly. One case of dislocation of the head of the os humeri, inwards under the pectoral muscle, of ten days' standing. The man, a sailor, had fallen from under his feet, on the deck of a vessel, at sea, and struck his shoulder. The dislocation was reduced by Dr. Norris, with the pulleys, after a large bleeding, and the administration of tartarized antimony; clavicle bandage afterwards applied, and the man was discharged, at his own request, the next day. One case of lacerated wound of the thumb, from the bursting of a gun, a portion of the muscle of the ball of the thumb being torn up, and exposed: poulticed, wound sloughing, and doing well. One case of oblique fracture of the upper third of the os femoris, caused by a fall from the third story of a house, in a fit of intoxication; symptoms of concussion of the brain were also present: the fracture was dressed with Physick's modification of Desault's splints; the man was very freely stimulated, and bladders of ice applied to the head; died in twenty-four hours. One case of lacerated wound of the hand, in a child, seven years of age, caused by the discharge of a gun, (loaded with powder and paper,) upon the hand, while on the muzzle of the gun. The wound extended between the metacarpal bones of the first and second fingers, dividing the palmar arch. There was no hæmorrhage, except from a small superficial vessel, which was taken up. The edges of the wound were drawn together by adhesive straps, and a dressing of dry lint applied. Cold applications were kept up by means of a syphon, and, at the present time, after a lapse of six days, the child has suffered very little pain, and has not had a bad symptom. The dressing has not been disturbed. One case of fracture of both bones of the leg, just above the ankle,—dressed with fracture-box, and cloths of lead-water. One case of contused wound of the thigh, caused by a piece of iron, plunged, red-hot, from the forge of a blacksmith, into the thigh. The iron passed to the depth of about three inches through the limb. The hæmorrhage that followed was inconsiderable, and the wound was dressed with a poultice, a tourniquet being loosely applied.

The cases, mentioned in the last number, are still under treatment.

